# Characterization of a distinct lethal arteriopathy syndrome in twenty-two infants associated with an identical, novel mutation in FBLN4 gene, confirms fibulin-4 as a critical determinant of human vascular elastogenesis

**DOI:** 10.1186/1750-1172-7-61

**Published:** 2012-09-03

**Authors:** Mahesh Kappanayil, Sheela Nampoothiri, Rajesh Kannan, Marjolijn Renard, Paul Coucke, Fransiska Malfait, Swapna Menon, Hiran K Ravindran, Renu Kurup, Muhammad Faiyaz-Ul-Haque, Krishna Kumar, Anne De Paepe

**Affiliations:** 1 Departments of Pediatric Cardiology, Amrita Institute of Medical Sciences and Research Centre, Kochi, India; 2 Department of Pediatric Genetics, Amrita Institute of Medical Sciences and Research Centre, Kochi, India; 3 Department of Radiology, Amrita Institute of Medical Sciences and Research Centre, Kochi, India; 4 Center for Medical Genetics, Ghent University, Ghent, Belgium; 5 SciGenome Labs Private Limited, Kochi, India; 6 Department of Pathology – Amrita Institute of Medical Sciences and Research Centre, Kochi, India; 7 Department of Pediatric Cardiology, Malabar Institute of Medical Sciences, Calicut, India; 8 Molecular Genetics Laboratory, King Faisal Specialist Hospital and Research Centre, Riyadh, Saudi Arabia; 9 Clinical Associate Professor, Pediatric Cardiology, Amrita Institute of Medical Sciences and Research Centre, Amrita Lane, Ponekkara Post, Kochi, Kerala, PIN:682041, India

**Keywords:** Arterial tortuosity, Fibulin-4 mutation, Aortic aneurysm, Vascular elasticity, Genetic vasculopathy, Mappila muslims, Founder effect, Cardiovascular imaging, Lethal mutation, Connective tissue disorder, Abnormal elastogenesis, Malabar

## Abstract

**Background:**

Vascular elasticity is crucial for maintaining hemodynamics. Molecular mechanisms involved in human elastogenesis are incompletely understood. We describe a syndrome of lethal arteriopathy associated with a novel, identical mutation in the fibulin 4 gene (*FBLN4*) in a unique cohort of infants from South India.

**Methods:**

Clinical characteristics, cardiovascular findings, outcomes and molecular genetics of twenty-two infants from a distinct population subgroup, presenting with characteristic arterial dilatation and tortuosity during the period August 2004 to June 2011 were studied.

**Results:**

Patients (11 males, 11 females) presented at median age of 1.5 months, belonging to unrelated families from identical ethno-geographical background; eight had a history of consanguinity. Cardiovascular features included aneurysmal dilatation, elongation, tortuosity and narrowing of the aorta, pulmonary artery and their branches. The phenotype included a variable combination of cutis laxa (52%), long philtrum-thin vermillion (90%), micrognathia (43%), hypertelorism (57%), prominent eyes (43%), sagging cheeks (43%), long slender digits (48%), and visible arterial pulsations (38%). Genetic studies revealed an identical c.608A > C (p. Asp203Ala) mutation in exon 7 of the FBLN4 gene in all 22 patients, homozygous in 21, and compound heterozygous in one patient with a p. Arg227Cys mutation in the same conserved cbEGF sequence. Homozygosity was lethal (17/21 died, median age 4 months). Isthmic hypoplasia (n = 9) correlated with early death (≤4 months).

**Conclusions:**

A lethal, genetic disorder characterized by severe deformation of elastic arteries, was linked to novel mutations in the FBLN4 gene. While describing a hitherto unreported syndrome in this population subgroup, this study emphasizes the critical role of fibulin-4 in human elastogenesis.

## Background

Vascular structure and integrity are determined by connective tissue elements, which include elastic fibers, collagen and several glycoproteins. Cardiovascular abnormalities resulting from inherited defects of many of these components are well known, e.g. Marfan syndrome (fibrillin-1) (MFS; MIM #154700), Williams-Beuren syndrome (elastin) (WBS; MIM#194050), vascular Ehlers-Danlos syndrome (type III collagen) (vEDS; MIM # 130050), and hereditary cutis laxa syndromes (elastin and fibulin-4 and 5) (ADCL1 and ARCL1A; MIM #123700, 219100). However, some of the most dramatic vascular manifestations observed occur in a group of rare disorders which includes Arterial Tortuosity Syndrome (ATS; MIM #208050), Loeys-Dietz Syndrome (LDS; MIM #609192) and Autosomal Recessive Cutis Laxa (ARCL) type I. ATS is an autosomal recessive disorder attributed to mutations in the SLC2A10 gene (chromosome 20q13) [GenBank:AF321240.1] [1]; LDS is an autosomal dominant disorder associated with heterozygous mutations in genes encoding the transforming growth factor beta receptors (TGFBR) 1 and 2 [GenBank:GU143401.1 and GU143402.1] [2]. It has been shown that perturbations in the TGF beta pathway contribute to the pathogenesis of some of the above-mentioned disorders. Despite the dramatic vascular phenotype and associated morbidities [3-6] ATS and LDS are reported to have a natural history permitting survival of several patients to adulthood [2,5-7]. ARCL Type I is characterized by cutis laxa, pulmonary emphysema, umbilical and inguinal hernias and gastrointestinal and vesico-urinary tract diverticuli, described in association with mutations of fibulin-5 [8] [GenBank:BC022280.1] and fibulin-4 [9] [GenBank:AF109121.1]. Though each of these disorders has characteristic phenotypic features [2,3,5,7-16], the dominating common features are dilatation, elongation and tortuosity of the large and medium sized arteries.

The large arteries (aorta and its branches, pulmonary arteries) are distinguished by their elasticity, which allows them to expand and recoil with cardiac contraction and relaxation, maintaining blood pressure and hemodynamics. This property is largely attributable to elastic fibers [17,18] present as sheets (lamellae) in the tunica media as well as, in sparser quantities, in internal elastic lamina and along with collagen in the tunica adventitia. Loss of elasticity can result in deformation in response to the constant hemodynamic stresses. While elastic fibers constitute only 2-3% of skin tissue, they contribute to nearly 50% of dry weight of elastic arteries [19]. Mature elastic fibers are composed of central amorphous elastin (derived from its soluble precursor, tropoelastin), surrounded by a scaffold of microfibrils. Microfibrils contain numerous proteins such as microfibrillar-associated glycoprotein, fibrillin and fibulin [17,18]. Recent studies have shown direct structural and regulatory roles of fibulin-4 and −5 in elastogenesis [20,21]. In addition, it has been postulated that mutations in fibulin-4 and −5 may impair elastogenesis through modulation of the TGF β signaling pathways [22]. The steps in elastogenesis and role of different molecules in formation and stabilization of elastic fibers are complex and incompletely understood [17].

This study characterizes a cohort of twenty-two infants from an identical ethno-social background with a lethal syndromic vasculopathy associated with a novel mutation in *FBLN4*, confirming fibulin-4 as a critical determinant in human elastogenesis.

## Results

### Clinical evaluation

Twenty-two patients (11 males, 11 females) were studied. All patients belonged to unrelated families from the same ethno-religious group (Muslim) hailing from the same geographical area, northern coastal (“Malabar”) belt of the southern Indian state of Kerala. Muslims of Malabar constitute a distinct community, commonly called “Mappilas”.

**Figure 1:**
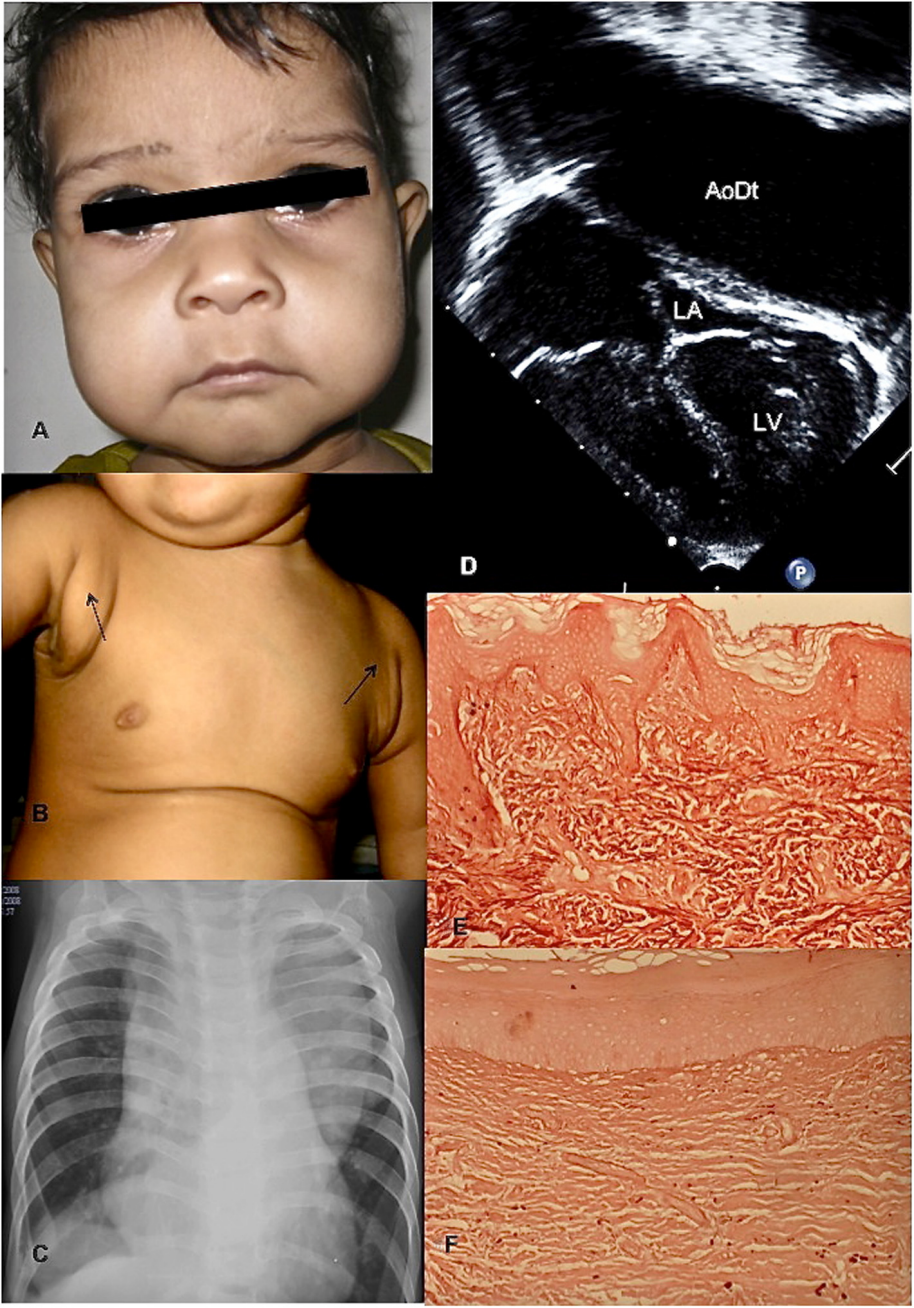
**Clinical features and histopathology.A** : Typical facial features – long philtrum thin upper lip, prominent eyes, broad forehead, sagging cheeks and lateral part of upper eyelids. **B** : Cutis laxa with redundant axillary skin folds (arrows). **C** : Chest roentgenogram (Antero-posterior view) – aortic enlargement evident as mediastinal widening. Dilated descending aortic shadow seen in right hemithorax just above the level of the diaphragm. **D** : Echocardiogram showing dilated descending thoracic aorta (AoDt) compressing upon the left heart structures (LA – left atrium, LV – left ventricle). [Figures  1A-1D belong to Proband 5]. **E** and   **F** : Orcein-stained skin biopsies from an age-matched control and proband 10 respectively, showing the abundant, dark stained elastic fibers in the control and severely deficient and fragmented elastic fibers in patient.

Table  1 shows the clinical profiles, family history and phenotypic features (Figure 1A, 1B). All patients had normal birth weights (median 2.96 kg). Two infants presented initially with an incidentally detected murmur, all others presented with cardiorespiratory distress. Six patients presented in neonatal period, two of them with severe cardiorespiratory distress and refractory shock within hours of birth. Vasculopathy was diagnosed at a median age of 1.75 months (1 day to 15.5 months). Antenatal history, routine fetal scans, and birth history were unremarkable in all except for one patient (proband 20) who was detected to have an excessively dilated aorta and pulmonary artery in third trimester fetal ultrasonogram. None of the other patients had undergone third-trimester fetal ultrasonography.

**Table 1:** Clinical and Phenotypic features

	**CLINICAL**					**PHENOTYPIC FEATURES**														
**S. No**	**Sex**	**Age at diagnosis (Months)**	**Birth order**	**Consanguinity**	**Family history**	**Cutis laxa**	**Hernia**	**Prominent eyes**	**Hypotonia**	**Micrognathia**	**Hypertelorism**	**Long philtrum, thin vermillion**	**Joint laxity**	**Abnormal ears**	**Broad forehead**	**Thin, long digits**	**Sagging cheeks**	**Hooded eyes**	**High arched palate**	**Seizures**
1	M	1.5	2	No	Yes	-	-	-	+	+	-	+	-	-	-	-	-	-	+	-
2	M	2	4	Yes	No	-	-	-	-	-	+	+	-	-	+	-	-	-	-	-
3	F	1.2	2	Yes	No	+	-	-	+	-	+	+	-	-	-	-	-	-	-	-
4	M	4	2	Yes	No	+	+	-	+	+	+	+	-	-	+	-	+	+	-	-
5	M	8	2	No	No	+	+	+	+	-	+	-	-	+	-	-	+	+	+	-
6	F	9	1	No	No	+	+	+	+	-	+	+	+	-	-	-	+	+	+	-
7	F	0.5	1	Yes	No	-	-	-	-	+	-	+	-	-	-	+	-	-	-	-
8	F	9	2	No	Yes	-	-	-	-	-	+	-	+	-	-	-	-	+	-	-
9	M	0.03	1	Yes	No	-	-	-	-	+	-	+	-	+	-	+	-	-	-	-
10	F	1.5	1	No	No	+	-	+	-	+	-	+	+	+	-	+	+	+	+	-
11	F	15	2	No	No	+	-	+	-	+	-	+	-	-	+	+	+	+	-	-
12	M	1.2	1	No	No	+	-	+	-	+	+	+	-	-	-	+	+	-	+	+
13	M	1	1	No	No	+	-	+	-	-	-	+	-	+	-	+	-	-	-	+
14	M	0.5	4	No	Yes	-	-	-	-	-	-	+	-	-	-	-	-	-	-	-
15	M	1.5	1	Yes	No	-	-	-	-	+	-	+	-	-	-	-	-	-	-	-
16	F	0.03	1	Yes	No	NA	NA	NA	NA	NA	NA	NA	NA	NA	NA	NA	NA	NA	NA	-
17	F	2	1	No	No	+	-	+	-	-	+	+	-	-	+	+	-	-	+	-
18	F	6	2	No	Yes	-	-	-	+	-	+	+	-	-	-	+	+	-	-	-
19	F	17	4	Yes	Yes	+	+	-	+	-	+	+	+	-	-	-	-	-	-	-
20	F	12	1	No	No	-	-	-	+	-	-	+	+	-	+	-	-	-	+	-
21	M	0.9	1	No	No	+	-	+	+	-	+	+	-	-	+	+	+	-	+	+
22	M	0.5	1	No	No	-	-	+	-	+	+	+	+	-	-	+	+	-	-	+

Eight of the patients were born out of third degree consanguineous marriages (between first cousins). Four families (probands 1, 14, 18, 19) had a history of death of a previous infant (age at death 4, 11, 12 and 24 months, respectively), all of whom were noted to have respiratory distress in association with at least one of the following - dilated great arteries (n = 2), loose skin (n = 2), or inguinal/abdominal hernia (n = 2). The mother of proband 18 had history of third trimester fetal loss from unknown cause.

The phenotype included variable combination of features, as listed in Table  1. The facial appearance was characteristic, with long philtrum and thin vermillion observed in 90%; other features being hypertelorism (57%), micrognathia (43%), prominent eyes (43%), sagging cheeks (43%), high arched palate (38%), hooded eyelids (29%), broad forehead (29%) and dysplastic/low set pinnae (19%). Cutis laxa with redundant skin folds was seen in 52%, most prominent in the axillary and anterior abdominal skin. Skin was hyperextensible in two patients. Thin long digits (48%), generalized hypotonia (43%), and herniae (19%) were the other common physical features observed. 67% (n = 14) of the patients had 5 or more of the listed features, 90.5% (n = 19) had 3 or more, while only 9.5% (n = 2) had less than 3.

Pulsatile arterial vascular swellings (measuring 1.5 cm-3 cm) were a striking finding in 8 patients, most prominently visible in the axilla, suprasternal, supraclavicular or submandibular locations (Additional file 1). No major skeletal abnormalities were noted in any patient.

Delay in attainment of gross motor milestones was observed in all infants older than four months, mostly in association with generalized hypotonia and debility.

### Cardiovascular imaging

**Figure 2:**
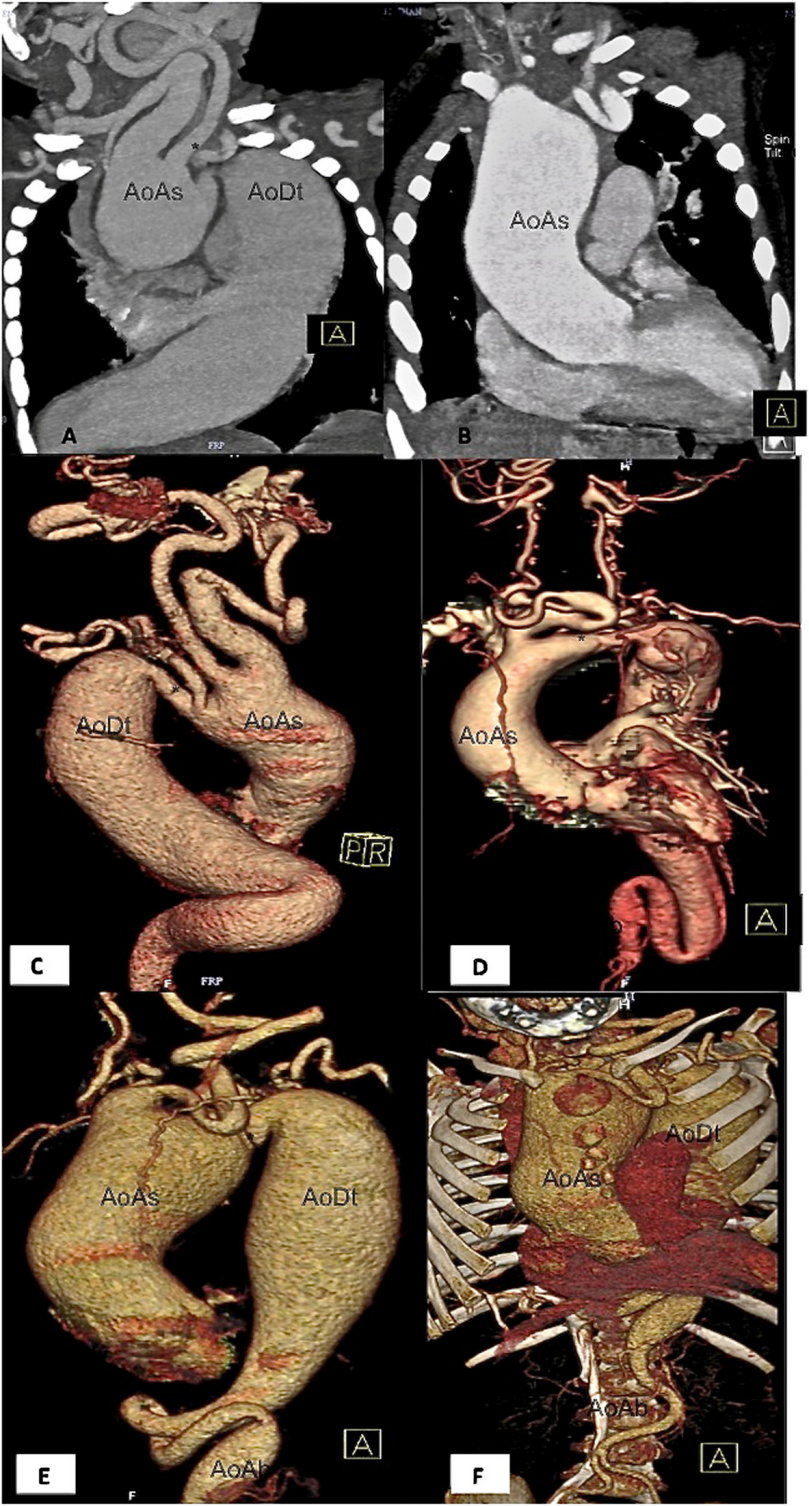
**Cardiovascular imaging.A**- **F** : 64-slice cardiac CT images showing characteristic aneurysmal enlargement and tortuosity of aorta and arch branches. Note the narrow aortic isthmus (*) ( **A**, **C**, **D**, **E**), abdominal aortic stenosis ( **D**- **F**). AoAs – Ascending Aorta, AoDt- Descending Thoracic Aorta, AoAb – Abdominal Aorta.

**Figure 3:**
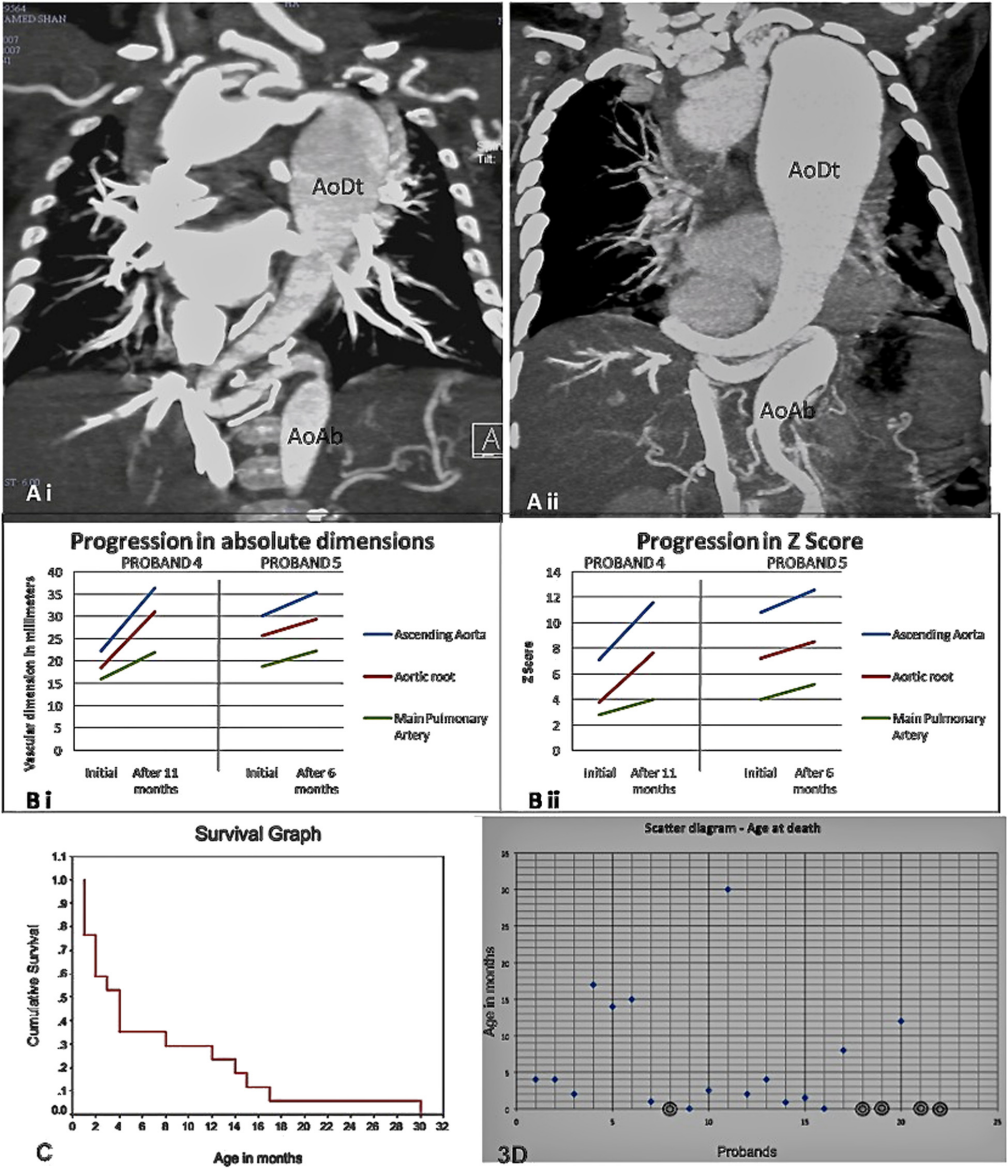
**Natural history and outcomes.Ai** and **Aii** : Serial 64-slice CT scans done in Proband 4 after an interval of 11 months showing progressive enlargement and tortuosity of descending aorta. **Bi** and **Bii** : Graphs showing the increase in the absolute dimensions and the respective Z-scores, of Ascending Aorta, Aortic Root and Main Pulmonary Artery in serial 64-slice CT scans in Proband 4 (at interval of 11 months) and Proband 5 (at interval of 6 months). **C**: Kaplan Meier Survival Graph for the homozygous mutant patients. **D** : Scatter diagram for the entire cohort, showing the ages at death. Currently surviving probands are encircled.

The chest roentgenogram showed mediastinal widening with aortic dilatation (Figure 1C). Lung volumes were compromised by the enlarged great arteries within the thorax. The findings on cardiovascular imaging are summarized in Table  2 and shown in Figures 1D, 2A-F, 3Ai, 3Aii and Additional file 2. Most striking in all patients were the impressive abnormalities of the aorta and pulmonary arteries, and their branches. While ascending and descending thoracic aorta, and the main pulmonary artery, were elongated and dilated in all, some sites like the aortic isthmus, the origins of the arch branches (branchiocephalic, carotid and subclavian arteries), abdominal aorta and its branches and the branch pulmonary arteries, were prone to stenosis. All patients who had peripheral pulmonary stenosis showed consequent right ventricular hypertension.

**Table 2:** cardiovascular manifestations

**Alteration in arterial dimensions**	**Absolute dimension (Range in mm)**	**Z score (Range)**	**Median Z score**
**Dilatation of aortic root**	14 to 30.4	+3.6 to +8.1	+4.6
**Dilatation of ascending aorta**	14 to 35.9	+4.6 to +12.1	+7.6
**Dilatation of proximal transverse arch**	6.5 to 29.1	−2.7 to +6.9	+3.4
**Narrowing of isthmus, coarctation**	1.8 to 7	−6.5 to −0.4	−2
**Dilatation of main pulmonary artery**	9.1 to 23.6	+0.5 to +7	+4.1
**Other Major findings**			
**Aorta & branches**			
Tortuosity and elongation of the arch branches – 22			
Origin stenosis of left subclavian artery – 5			
Origin stenosis/atresia of left common carotid artery – 4			
Origin stenosis of bilateral carotid arteries – 2			
Elongation and tortuous folding of thoracic aorta at diaphragm – 18			
Coeliac artery stenosis - 4			
Abdominal aortic dilatation – 6			
Abdominal aortic stenosis - 8			
Stenosis of superior mesenteric artery – 2			
**Pulmonary arteries**			
Right pulmonary artery stenosis – 8			
Left pulmonary artery stenosis – 2			
Early branching of the right pulmonary artery – 7			
**Other findings**			
Ventricular hypertrophy – 15			
Aortic regurgitation (mild) – 10			
Mitral regurgitation (mild) - 7			
Tracheal compression – 6			
Esophagial compression - 7			
Cerebral arterial elongation/tortuosity – 9 (of 11patients scanned for intracranial arteries)			
Cerebral infarctions - 3			

Table  2 summarizes the findings on cardiovascular imaging (echocardiography, 64-slice CT scan / MRI).

Parental cardiovascular evaluation by echo or MRI was normal in all the nine cases studied.

Cranial CT revealed elongation and tortuosity of cerebral arteries in nine patients, and signs of cerebral ischemia in three of these. The latter three had stenosis of the origin of common carotid arteries. Retinal vessels were found to be prominent but non-tortuous in both the patients subjected to retinal examination (probands 5 and 6).

Progression of the vascular lesions was documented in CT scans repeated after 11 and 6 months respectively for probands 4 and 5 as shown in Figures 3-Ai, Aii, Bi, Bii. Tracheal and esophageal compression observed on the CT correlated with their symptoms of positional (supine) dyspnea and dysphagia.

### Histopathology

Hematoxylin-eosin and Orcein staining of a skin biopsy of proband 10 showed deficient and fragmented elastic fibers in the subcutaneous layers, as compared to an age-matched control (Figure 1E,F).

### Molecular genetics

In the absence of a precise molecular diagnosis in the initial period of the study, patients were assigned a provisional clinical diagnosis of Arterial Tortuosity Syndrome [23] on the basis of overlapping physical and vascular phenotype. Therefore, initially molecular analysis of the SLC2A10 gene was performed, but no disease-causing mutation was identified. Patients did not present with Loeys-Dietz syndrome strictly speaking, nevertheless mutations in the TGFBR1/2 genes were excluded by direct sequencing of both genes. Subsequently, direct sequencing of the FBLN4 gene revealed an *identical* homozygous c.608A > C (p. Asp203Ala) mutation in exon 7 in all patients except for the 8^th^ proband who showed compound heterozygosity for this mutation with a c.679C > T (p. Arg227Cys) mutation in the same calcium binding epidermal growth factor (cbEGF) domain. Parental DNA of 19 probands were tested (both parents of 12 probands , and only maternal DNA of 7 probands). All parents tested were heterozygous for the same c.608A > C (p. Asp203Ala) mutation, except for the father of the compound heterozygote who did not bear any *FBLN4* mutation, indicating that the p. Arg227Cys occurred *de novo* (with proven paternity). The presence of the mutations were excluded in 200 control alleles. In order to determine a common ancestry, we haplotyped several polymorphic markers surrounding the FBLN4 gene (D11S4205 – 0.2 Mb – D11S1883 – 2 Mb – *FBLN4 –* 1.9 Mb – D11S1889 – 0.1 Mb – D11S4155 – 1.35 Mb – D11S4113 – 0.5 Mb – D11S4095). These analyses showed a shared haplotype for the following three markers: D11S1889, D11S4155, and D11S4113 in all probands.

### Outcome and natural history

All patients were managed with symptomatic and supportive measures, using digoxin and diuretics where indicated, respiratory care, nutritional supplements and physiotherapy. Two patients (probands 1 and 2) received propranolol (2 mg/kg/day q 12 hours) and all the remaining homozygous mutant patients received losartan (0.5-1.5 mg/kg/day q 24 hours). Among the homozygous mutant patients (n = 21), 76.2% (n = 17) died, median age of death being 4 months (36 hours-30 months, average 6.9 months) (Figure 3C-D), including 4 neonatal deaths. Probands 9 and 16 had the shortest survival, dying at 36 hours and 48 hours of life respectively, following intractable cardiorespiratory failure and shock soon after birth. The other two neonatal deaths (probands 7 and 14) occurred at 15 days and 28 days, respectively, due to rapidly worsening cardiorespiratory failure. The remaining homozygous mutant patients also showed progressive worsening of symptoms; tachypnea and chest indrawing especially in the supine position, deterioration during respiratory infections, feeding difficulties, failure to thrive, increasing skin laxity (in patients who had cutis laxa initially), generalized hypotonia, and delayed motor development. Four patients (probands 12, 13, 21, 22) developed generalized seizures on follow-up (in early infancy) and required anti-convulsants; all four had evidence of stenotic lesions in common carotid artery origins and/or in the smaller cerebral vessels (CT angiography). Only 6 of the 21 homozygous mutants survived beyond first year of life (probands 4,5,6,11,18,19), three of whom subsequently died between 14–17 months of age (probands 4,5,6). Proband 11 was the sole survivor beyond 2 years, dying eventually at 30 months. All patients who survived beyond first year of life showed preference for a lateral position while sleeping and leaning forward position while feeding, suggesting airway and esophageal compression (also demonstrated in CT/MRI). Arterial pulsations noted in axilla, neck and submandibular regions became more prominent on follow-up; newer pulsatile masses appeared in three patients on follow-up. The four surviving homozygous mutants, probands 18, 19, 21, 22 are currently aged 19, 22, 2 and 2 months respectively. All four are receiving Losartan (1.5 mg/kg/day), digoxin and frusemide. Proband 21 developed hemopericardium and cardiac tamponade with intracranial bleed at 55 days of age requiring emergency pericardiocentesis and blood transfusion. CT Angiogram failed to reveal the site or cause of the pericardial and intra-cranial hemorrhage; coagulation parameters were normal. All the other three surviving patients have history of recurrent hospitalization for intermittent worsening of respiratory symptoms especially during respiratory infections, improving with medications. Proband 8, bearing the compound heterozygous mutations has remained asymptomatic and is currently 7 years old.

Except for proband 17, none of the deaths occurred at our center; events surrounding death were noted by interviewing the care givers. Six deaths were precipitated by respiratory infections, and one was preceded by multiple generalized seizures. Terminal events in all were worsening cardiorespiratory symptoms and general debility. Vascular rupture as a terminal event could not be established in any of these patients, however the possibility cannot be excluded. None of the families consented for autopsy.

Aortic isthmic hypoplasia (Z score ≤ −2, range −2.1 to −6.49, n = 9) correlated with early clinical presentation (≤ 2 months) and early death (≤ 4 months) (p value <0.0001).

Antenatal counselling was offered to the mother of proband 1 during her subsequent pregnancy. Fetal echocardiogram and anomaly scans at 17 weeks of gestation were normal, but amniocentesis and mutation analysis revealed fetal homozygosity for the *FBLN4* mutation. The pregnancy was terminated at insistence of the family at 18 weeks. Autopsy and post-mortem aortogram did not reveal obvious arterial abnormalities. Fetal skin biopsy showed near-absence of elastic fibers.

## Discussion

This study describes the spectrum of clinical features, natural history, and molecular genetics of a disorder resulting from a novel mutation in the gene encoding the fibulin-4 protein in a unique population subset.

**Figure 4:**
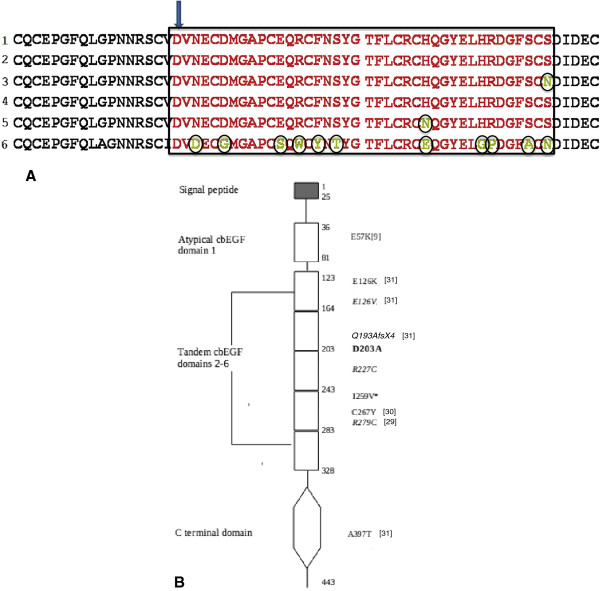
**Sequence alignment of fibulin-4 and location of mutations.A**: Multiple sequence alignment of fibulin-4 protein sequence from (1) *Homo sapiens,* (2) Pan troglodytes*, (3) Canis lupus familiaris*, (4) *Bos taurus,* (5) *Mus musculus,* (6) Danio rerio ; sequences from species marked * were computationally predicted. cbEGF4 sequence is enclosed in the rectangular box. Substitutions within the domain are encircled. Arrow marks the position of aspartate 203 in human fibulin-4. Conservation of amino acid identity of this aspartate across species and its sequence position at start of the cbEGF4 domain, indicates its functional significance. All patients had identical missense mutation at this crucial position. **B** : Schematic representation of domains, based on Swiss-Prot annotation [Swiss-Prot:O95967]. The types of domain are listed on the left. Amino acid position in fibulin 4 sequence is noted at start and end positions of the protein, the atypical cbEGF domain and the region containing 5 tandem cbEGF domains. Mutations listed on the right are found in human disease with the exception of I259V*. The D203A mutation (bold) is the one found in all patients in our cohort. Mutations shown in italics occurred in compound heterozygotes. References are shown in brackets.

Fibulins constitute a recently recognized family of seven extracellular glycoproteins that interact with many extracellular matrix proteins including components of basement membranes and elastic fibers [24]. They are widely distributed throughout the body, especially in elastic-fiber rich tissues (aorta, lungs etc.). Fibulins contain contiguous calcium-binding epidermal growth factor-like domains (cbEGF) and a characteristic C-terminal fibulin domain [25] (Figure 4B. Fibulins-4 and −5 are implicated in elastogenesis [21,22,26].

Critical importance of fibulin-4 in elastogenesis has been shown in animal experimental models. Fibulin-4 *knock-out* mice showed severe vascular and lung defects and perinatal lethality due to abolition of elastogenesis [27]. Suppression of elastogenesis in fibulin-4 *knock-down* mice also resulted in vascular tortuosity and aneurysms resembling human arterial tortuosity syndromes [22,28]. The role of fibulin-4 in human elastogenesis has been recently demonstrated experimentally in human foreskin fibroblast model [20]. However, reports of mutations in *FBLN4* resulting in clinical disease in humans are rare. Hucthagowder et al. [9] identified a missense mutation (c.169 G > A; p.E57K) in *FBLN4* in a child with multiple fractures, cutis laxa, vascular tortuosity and aneurysms, emphysema, inguinal and diaphragmatic hernia and joint laxity. Compound heterozygous mutations ((c.835C > T (p.R279C) / c.1070_1073dupCCGC) in *FBLN4* causing a severe phenotype including cutis laxa and aortic aneurysm and tortuosity resulting in death at 27 days age was reported by Dasouki et al. [29]. Hoyer et al. reported a homozygous missense mutation (p. Cys267Tyr) in a female neonate with fetal overgrowth and oligohydramnios, arachnodactly, contractures, cutis laxa, microcephaly, spina bifida and extreme perinatal bradycardia leading to death [30]. This group of *FBLN4* mutation-positive patients was doubled by the identification of 2 homozygous (c.376 G > A; p. Glu126Lys, and c.1189 G > A; p. Ala397Thr) and 1 compound heterozygous mutations (c.377A > T: p. Glu126Val, and c.577delC; p. Gln193Ser fs X12) in three patients by Renard et al. [31]. These patients were clinically characterized by major cardiovascular defects (aortic aneurysms, arterial tortuosity, and stenosis) and a marked absence of severe skin involvement. All mutations in *FBLN4* discussed here are depicted in Figure 4B.

Mechanisms whereby fibulin-4 absence or deficiency results in aneurysm formation are not established. While Chen Q et al. [20] and Choudhary et al. [21] postulated direct structural role for fibulin-4 in elastic fiber assembly, Hanada et al. [22] postulated TGF beta upregulation as possible mechanism in the murine model. Renard et al. provided the first evidence of altered TGF beta signaling in humans with *FBLN4* mutations [31]. Huang et al. observed that while *Fbln4* null mice develop lethal aortic aneurysms, deficiency of other elastic elements like fibulin-5 and elastin were not associated with as severe aneurysm formation or lethality. They postulated a dual role for fibulin-4, in elastic fiber formation, as well as terminal differentiation and maturation of SMC (smooth muscle cells) in aortic wall [32].

We describe 22 new patients with an identical novel mutation in *FBLN4*, with severe vascular phenotype and lethal outcomes, providing the largest clinical evidence so far, for vital role of fibulin-4 in human elastogenesis.

The first mutation identified was homozygous single base substitution (c.608A > C) in 21/22 probands, resulting in p. Asp203Ala substitution which affects the highly conserved DVNE consensus sequence of the fourth cbEGF domain (Figure 4A). The consensus sequence is critical in binding calcium ions, which are essential for the structural and binding properties of the fibulin-4 protein. The second mutation, an argine 227 to cysteine substitution, of the compound heterozygote patient (proband 8) results in substitution of a positively charged arginine to a disulfide containing cysteine which may foster incorrect disulfide bond formation or interfere with other charge interactions important for protein structure stability, or functional interactions potentially leading to altered secretion into the extracellular matrix. The causal nature of the mutations is further supported by the following facts: (1) the mutations were not present in 200 control alleles, (2) they affected highly conserved amino acids and nucleotides (Figure 4A), (3) by its *de novo* occurrence (p. Arg227Cys), (4) the mutations are predicted to have a pathogenic effect based on *in silico* prediction software (SIFT: deleterious (score 0.00); Polyphen2: probably damaging (score 1.000); Align GVGD: class65 (GV 0.00 – GD 125.75)), and (5) the mutations lead to an amino acid change which results in a moderate (p. Asp203Ala, Grantham score 126, acidic to hydrophobic amino acid) to large (p. Arg227Cys, Grantham score 180, basic to hydrophobic amino acid) physicochemical difference.

**Figure 5:**
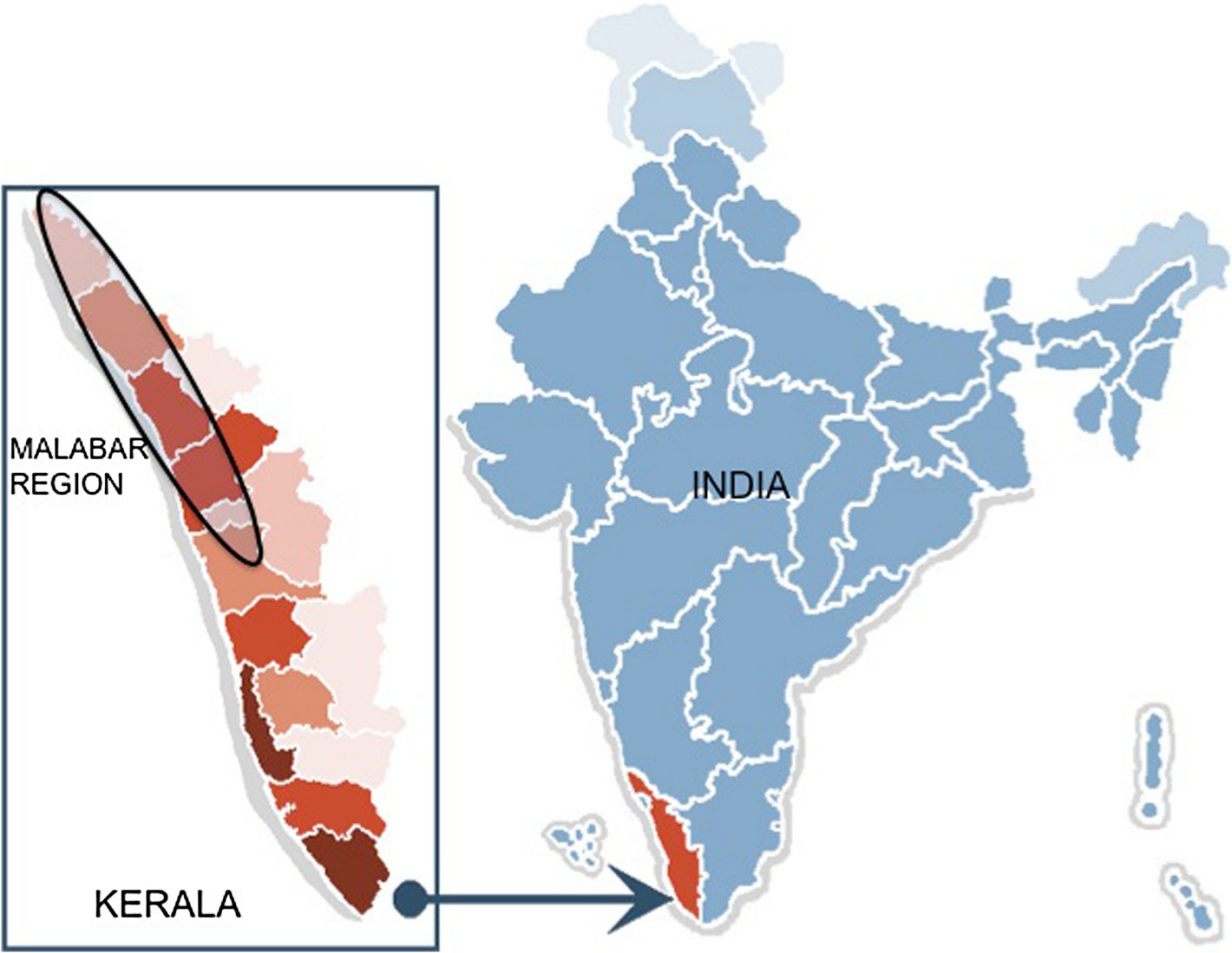
**Map of India.** Political map of India showing the state of Kerala (panel inset) and the Malabar region (encircled). Source : Wikimedia.

The unique social background of our patients is intriguing. The northern coast of Kerala (Malabar) has a population of about 17.5 million, and a large proportion of the Muslims in Kerala (about 24%) [33] (Figure 5). The ports of Malabar were historically important as hubs of sea trade in ancient and medieval India. Mappilas of Malabar are historically regarded as the first Muslims in India, many of them originally descended from Arab settlers who brought the religion to this region as early as 7^th^ century AD [34]. Consanguineous marriages are common among this distinct sub-population. Most marriages are arranged within the Mappila community even in the current era. The presence of a shared haplotype including the FBLN4 gene among the families in our cohort strongly suggests a common ancestry for all probands. Limited size of the shared haplotype block (max 5.85 Mb), favors the existence of a relatively old founder. The significance of this unique historical/ethnic background needs further evaluation. No major studies exist on the genetic profile or diseases specific to this community. The finding that all parents tested (except father of proband 8) in this cohort were heterozygous for the same mutation, bearing normal phenotype, suggests that the mutation is not lethal in its heterozygous state and therefore allows transmission.

All homozygous mutant patients showed early presentation and high lethality. Only 4/22 homozygous mutants currently survive. Correlation of aortic isthmic hypoplasia with early clinical presentation and death implicates severity of coarctation as an important predictor of outcomes. Neonatal presentation and death in four, as well as the 3^rd^ trimester antenatal detection of vascular dilatation in one patient implies presence of advanced vascular lesions at birth in at least some of these patients. Although the terminal event in most appeared to be worsening cardiorespiratory failure, inability to subject these patients to autopsy limits our understanding. Notably, proband 8 bearing a compound heterozygous mutation is surviving with no major symptoms despite having vascular abnormalities similar to the homozygotes (Z scores: aortic root, +8.14; ascending aorta, +12.14; isthmus, -1.55; MPA, +3.41). Even though survival may be linked to absence of severe coarctation, the overall asymptomatic status and longevity of this patient as compared to the homozygous mutant patients with similar aortic isthmic Z-scores sets this patient clinically apart. There may be a role for factors other than just physical deformation of arteries and coarctation, in determining mortality and morbidity.

While the phenotypic features overlapped with those of ATS, LDS and ARCL Type I, severe vascular abnormalities were the dominating feature in all. Aortic dilatation was more impressive than pulmonary artery dilatation, and more rapidly progressive, reflecting greater hemodynamic stresses within the aorta. Aortic isthmus was relatively narrow in all patients (median Z score −2) and in sharp contrast to the ascending aorta (median Z scores: +8.6) and descending aorta. Obstructive lesions were also noted in the branch pulmonary arteries, origins of arch branches and abdominal aorta. Reasons for this pattern of differential vascular growth is unclear and could point to differences in elastic fiber content at different sites. Ventricular hypertrophy seen in most patients was probably secondary to the obstructive lesions (coarctation, peripheral pulmonary stenosis).

Interestingly, in the only case where a fetal molecular diagnosis was established through amniocentesis, no arterial deformation was observed on prenatal ultrasonogram (17 weeks) or, fetal autopsy (18 weeks). Family opted for termination of pregnancy in view of two previous infant deaths (both at 4 months of age), one of whom was a proven homozygous mutant. Although in the absence of a large study of controls in this specific population, it is difficult to prove that all homozygotes for the mutation would have lethal vasculopathy, the normal vascular phenotype in this fetus could indicate that early fetal diagnosis is dependent on mutation analysis. It could also be an indicator of progressive nature of the vasculopathy through fetal life, possibly in response to hemodynamic stresses and other molecular factors. It has previously been demonstrated in fetal calf model that while elastic fibers at term are large and predominantly made up of central amorphous elastin, those in very early fetal life consist of only microfibrils; progressive accumulation of elastin occurs with advancing gestation [35]. It is therefore likely that vascular deformation occurring due to abnormal elastic fiber formation would become manifest only in later pregnancy.

## Conclusions

There exists a hitherto undescribed syndrome of arterial dilatation and tortuosity with lethal outcomes in the Malabar Mappila community, related to a novel mutation in the FBLN4 gene. The actual disease burden in this community is likely to be much higher and needs extensive studies. Additionally, it has been once again shown that fibulin-4 is critical to human elastogenesis, vascular integrity, and survival.

## Methods

### Patient population

This was a collaborative study conducted at Amrita Institute of Medical Sciences, Kochi, Kerala, India, and Center for Medical Genetics, Ghent, Belgium, between August 2004 and June 2011. Informed consent was obtained from families of all patients involved in the study.

The study group comprised 22 patients identified to have dilatation and tortuosity of the aorta and its branches on echocardiography and/or radiological studies, during the study period. All patients underwent detailed clinical, phenotypic and socio-demographic characterization, specialized cardiovascular imaging, molecular genetic studies, histopathological studies (wherever feasible), and clinical follow-up for outcomes. Phenotypic features were described by a clinical geneticist. Of three neonates, where sickness and death precluded clinical examination by the geneticist, phenotype was recorded from clinical photographs in two; no phenotypic details were available for the third neonate (proband 16). Retinal examination was done for two patients (probands 3 and 4).

### Cardiovascular imaging

Cardiovascular imaging was done using echocardiography and either 64-slice computed tomography (CT) (n = 20) or magnetic resonance imaging (MRI) (n = 2). Dimensions of the aorta and the pulmonary arteries were measured on CT or MRI at standard pre-defined locations. Z-scores were derived for selected measurements (aortic root at the sinuses of Valsalva, ascending aorta at the level of right pulmonary artery, isthmus just beyond the subclavian artery and main pulmonary artery) using published data sources [36-38]. Cerebral arteries were screened by CT scan in the eleven patients most recently clinically evaluated. Parental cardiovascular imaging was done by echocardiography in two, and cardiac MRI in seven cases.

### Molecular analyses

Molecular genetic studies were carried out using DNA isolated from the peripheral blood leucocytes from all patients, and one or both parents of 19 patients. Studies included exclusion of mutations of *SLC2A10* (associated with ATS) and *TGFBR1* and *TGFBR2* (associated with LDS) in the first 3 patients (proband 1–3) recruited. Direct sequencing of *FBLN4* was done for all patients. DNA from 100 individuals was used as control. Haplotyping around the FLBN4 gene was performed by PCR amplification of the following markers: D11S1205, D11S1883, D11S1889, D11S4155, D11S4113, D11S4095. Fragment analysis was done on ABI 3730XL.

### Histology

A skin biopsy was obtained from one patient (proband 10) from the anterolateral aspect of the right thigh and studied after hematoxylin-eosin, and Orcein staining using standard histopathological techniques.

### Natural history

Natural history was studied through clinical follow-up. CT scan was repeated for two (probands 4 and 5, 11 and 6 months after their respective initial CT evaluations) to determine rate of progression of arterial dilatation. Symptoms and circumstances surrounding deaths during follow-up were recorded.

## Competing interests

The authors declare that they have no competing interests.

## Authors’ contributions

MK and SN performed the clinical workup of the patients described in this report, participated in study design and coordination, and drafted the manuscript. RK performed and compiled the radiological imaging (CT and MRI) of all the patients. MR, PC and FM carried out the molecular genetic studies and helped to draft the manuscript. SM provided bioinformatics inputs and helped to draft the manuscript. HKR described the histopathogical features on tissue samples from patients. RPK contributed to clinical data acquisition. MFUH contributed to initial molecular genetic studies. KK participated in study design and critically revised the manuscript. ADP participated in the design and coordination of the study and critically revised the manuscript. All authors read and approved the final manuscript.

## Supplementary Material

Additional file 1 **Pulsatile arterial mass.** This video shows a large pulsatile mass in the left submandibular region in Proband 5. click here for file

Additional file 2 **Echocardiogram (axial view).** This video shows the dilated descending aorta compressing upon the heart posteriorly, in Proband 4. click here for file
